# A Multifunctional Tissue‐Engineering Hydrogel Aimed to Regulate Bacterial Ferroptosis‐Like Death and Overcoming Infection Toward Bone Remodeling

**DOI:** 10.1002/advs.202309820

**Published:** 2024-06-19

**Authors:** Renjie Lu, Zhiyuan Luo, Yuanyuan Zhang, Jiahao Chen, Yang Zhang, Chi Zhang

**Affiliations:** ^1^ Department of Orthopedic Surgery, Shanghai Institute of Microsurgery on Extremities Shanghai Sixth People's Hospital Affiliated to Shanghai Jiao Tong University School of Medicine 600 Yishan Road Shanghai 200233 China; ^2^ Nanomedicine and Intestinal Microecology Research Center, Shanghai Tenth People's Hospital, School of Medicine Tongji University 301 Yanchang Road Shanghai 200072 China; ^3^ Precision Medicine Center Taizhou Central Hospital 999 Donghai Road Taizhou Zhejiang 318000 China

**Keywords:** bacterial ferroptosis, immunomodulation, macrophage polarization, tissue repair, tissue‐engineering hydrogel

## Abstract

Infection is the most common complication after orthopedic surgery and can result in prolonged ailments such as chronic wounds, enlarged bone defects, and osteomyelitis. Iron, which is essential for bacterial metabolism and immune cell functions, is extremely important. Bacteria harness iron from nearby cells to promote biofilm formation, ensuring their survival. Iron deficiency within the infection microenvironment (IME) consequently hampers macrophage function, enabling further dissemination of the infection and hindering macrophage polarization to the M2 phenotype. Therefore, a novel approach is proposed to regulate macrophage polarization, aiming to restore the inflammatory immune environment. A composite hydrogel derived from natural polymers is developed to address infections and manage iron metabolism in macrophages. This IME‐responsive hydrogel, named FCL‐ECMH, is synthesized by encapsulating vermiculite functional core layers within a decellularized extracellular matrix hydrogel. It is noteworthy that FCL‐ECMH can produce reactive oxygen species within the IME. Supplementary photothermal treatment enhances bacterial iron uptake, leading to ferroptosis‐like death. This process also rejuvenates the iron‐enriched macrophages around the IME, thereby enhancing their antibacterial and tissue repair functions. In vivo experiments confirmed the antibacterial and repair‐promoting capabilities of FCL‐ECMH, indicating its potential for clinical applications.

## Graphical abstract

Herein, a multifunctional tissue‐engineering hydrogel (FCL‐ECMH) is presented for the treatment of tissue infection using a novel dual approach strategy involving promoting bacterial ferroptosis‐like death and regulating macrophage polarization. The synergistic effect contributes to the outstanding therapeutic efficacy of FCL‐ECMH against musculoskeletal infections, providing a new option for the clinical treatment of refractory musculoskeletal infections.

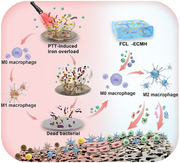

## 1 Introduction

Musculoskeletal infections, characterized by bacterial colonization and proliferation in the infected tissue, are common and severe complications of orthopedic surgery. These infections occur when bacteria aggregate and adhere to wounds or implants, leading to ineffective antibiotic treatment. The persistence and non‐healing of orthopedic infections are primarily caused by localized bacterial invasion and opportunistic dissemination.^[^ ^1^ ^]^ The microenvironment at the infection site plays a significant role in promoting the transformation of monocytes into osteoclasts, resulting in prolonged fracture healing and chronic osteomyelitis.^[^ ^2^ ^]^ Clinically, surgeons often resort to two‐stage surgery for these patients; however, this approach still faces challenges, such as lengthy treatment cycles and uncertain therapeutic effects. Bacterial infection hinders the immune response against biofilm formation in the microenvironment using exotoxins and quorum sensing systems, and depriving immune cells of iron required to evade the immune system. Consequently, the lack of immune surveillance leads to persistent infections and severe sepsis.^[^ ^3^ ^]^ Therefore, traditional antibacterial treatments alone have a minimal effect on controlling or eradicating stubborn bacterial infections without immune support. Hence, novel clinical treatments that combine anti‐inflammatory, reparative, and immune‐enhancing therapies are urgently required.^[^ ^4^ ^]^

Over the past few years, the application of nanomaterials for fighting bacteria has gained significant attention.^[^ ^5^ ^]^ However, the presence of dense and intricate biofilms, which possess strong resistance to antibiotics, heat, and oxidative stress, hinders the effectiveness of single nano‐biomaterial treatments, such as photothermal therapy (PTT), magnetic hyperthermia therapy (MHT), and sonodynamic therapy (SDT), in eliminating bacteria. Nonetheless, numerous studies have demonstrated that localized hyperthermia can stimulate immune cell function in infected regions.^[^ ^6^ ^]^ It is suggested that the generation of heat by light in the vicinity of the infection can trigger a death process similar to ferroptosis in bacteria, and the rejuvenation of neutrophils surrounding the IME can restore the suppressed anti‐biofilm function.

Macrophages, as essential cells of the innate immune system, play a crucial role in regulating the immune response within the IME.^[^ ^7^ ^]^ In response to the IME, macrophages can polarize into various phenotypes. During the initial pro‐inflammatory phase, classically activated macrophages (M1) are predominant; however, the shift from inflammation to proliferation is primarily controlled by alternatively activated macrophages (M2). However, in the presence of local hypoxia and immune dysfunction, conversion from the M1 to M2 phenotype is significantly suppressed during the inflammatory stage. Therefore, it is crucial to control the immune environment to expedite the resolution of the inflammatory phase by shifting the polarization of macrophages toward the M2 phenotype.^[^ ^8^ ^]^

Recently, there has been a significant focus on hydrogels as wound dressings to promote tissue regeneration because of their porous 3D structures and efficient swelling capabilities. They are capable of absorbing exudates to maintain a moist environment and decrease local inflammation.^[^ ^9^ ^]^ Additionally, they have been utilized as delivery systems for loading biofunctional components or silver nanoparticles (AgNPs), aiding wound healing.^[^ ^10^ ^]^ Currently, the primary approach for enhancing tissue healing by controlling M2 polarization involves the use of hydrogels containing antibiotics, cytokines, stem cells, miRNAs, extracellular vesicles, and growth factors. Nevertheless, these approaches are still not adequate because of the need for advanced manufacturing, drug resistance, unsustainable outcomes, and exorbitant expenses.^[^ ^11^ ^]^ In addition, regulatory mechanisms are rarely documented. Therefore, it is difficult to create a hydrogel that possesses inherent immunoregulatory capabilities to enhance tissue healing, and it is necessary to investigate additional mechanisms.^[^ ^12^ ^]^

In this study, we employed a universal wet chemical exfoliation technique utilizing alkali etching to selectively extract ultrathin and biocompatible functional core layers (FCL) (MgO and Fe_2_O_3_) sandwiched between two identical tetrahedral layers (SiO_2_ and Al_2_O_3_) from vermiculite, a Chinese patented medication known for its anti‐inflammatory properties.^[^ ^13^ ^]^ Following PEGylation using amine‐functionalized polyethyleneglycol (PEG‐NH_2_), the extracted FCL can effectively bind to an acellular matrix hydrogel. The iron (III) oxide component of the FCL facilitated Fenton reactions with hydrogen peroxide to produce hydroxyl radicals (·OH), which mediated chemodynamic therapy (CDT). This effect was significantly amplified when exposed to 808 nm lasers.^[^ ^14^ ^]^ Furthermore, FCL can effectively regulate the biofilm microenvironment by producing O_2_ and depleting glutathione (GSH), thereby reducing the hypoxic conditions and antioxidant capacity of the microenvironment.^[^ ^15^ ^]^ Additionally, FCL demonstrated high efficiency in converting light into heat during PTT when exposed to 808 nm laser radiation, resulting in a significant synergistic effect and enhanced CDT/PTT.^[^ ^16^ ^]^ Moreover, from a biophysical perspective, it is essential to apply scaffolds with favorable physical properties, such as mechanical adaptability and the ability to facilitate cell migration, to sites of tissue damage for successful tissue regeneration. Hydrogels have demonstrated significant promise for creating microenvironments that are relevant to the extant physiology, promoting cell growth, and aiding tissue regeneration. To enhance the strength and controlled‐release capabilities of FCL in bone and tissue structures, we integrated FCL with a decellularized extracellular matrix hydrogel (ECMH) to produce a versatile hydrogel, named FCL‐ECMH. This innovative hydrogel holds potential as a viable treatment option for tissue infections.^[^ ^17^ ^]^

**Scheme 1:**
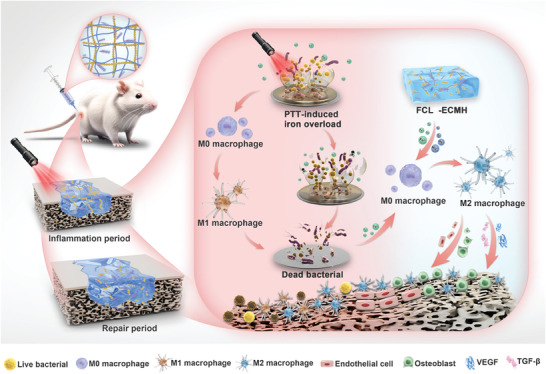
Schematic of the mechanism of the FCL‐ECMH induced infected tissue regeneration through regulating the macrophage polarization in healing process.

Thus, as illustrated in **Scheme** **1**, a combination of hydrogel with immunoregulatory properties was created as a dressing to enhance the healing process by manipulating the immune surroundings.^[^ ^18^ ^]^ FCL‐ECMH was successfully synthesized using a single‐step photopolymerization technique involving FCL and a decellularized hydrogel derived from the extracellular matrix.^[^ ^19^ ^]^ The morphology and mechanical performance of the hydrogel were assessed before and after the incorporation of FCL. Several in vitro studies have been conducted to assess cytocompatibility, antioxidative properties, antibacterial efficacy, angiogenic potential, and macrophage polarization of the hydrogel.^[^ ^20^ ^]^ The study also investigated FCL‐ECMH in inducing polarization of macrophages, specifically focusing on the mechanism of M2 polarization in macrophages. The impact of FCL‐ECMH on wound healing has been examined using various models simulating infectious diseases.^[^ ^21^ ^]^ In summary, a novel hydrogel with immunoregulatory properties was effectively synthesized, demonstrating various functions, including antibacterial activity, resistance to oxidation, promotion of angiogenesis, and enhancement of M2 polarization. FCL‐ECMH has potential applications in orthopedic infections.^[^ ^22^ ^]^

## 2 Results and Discussion

### 2.1 Synthesis and Characterization of FCL and FCL‐ECMH

**Figure 1:**
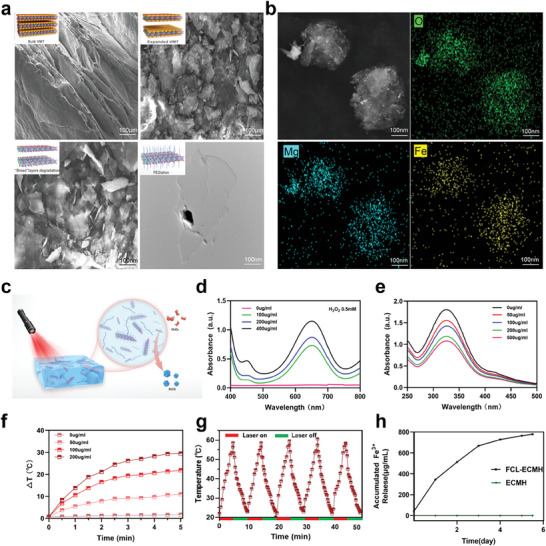
Characterization of FCL and FCL‐ECMH. a) Scanning electron microscopy (SEM) images and transmission electron microscopy (TEM) images of FCL during their preparation: upper left—bulk vermiculite (scale bar 100 µm), upper right—vermiculite particles after calcination (scale bar 100 µm), bottom left—vermiculite particles after NaOH etching (scale bar 100 nm), and bottom right—FCL (scale bar 100 nm). b) SEM‐EDX mapping images of FCL (scale bar 100 nm for all panels). c) Schematic of the mechanism of the FCL‐ECMH. d) UV–vis absorption spectra recording TMB oxidation over FCL‐PEG solutions with different concentrations. e) GSH degradation profile treated with FCL at different concentrations. f) The photothermal conversion performance of FCL (0–200 µg mL^−1^) irradiated by an 808 nm laser (2 W cm^−2^). g) Heating and cooling curves of the FCL. h) Iron ions can be released from the FCL‐ECMH simultaneously at a moderate rate (mean ± SD, n = 5). Three times was repeated each experiment independently with similar results in all these characterization figures.

Following ball milling, calcination, etching with NaOH, and sonication, the ultrathin central layer of vermiculite was effectively separated. Uniform vermiculite microparticles, ≈700 nm in diameter, were obtained after wet grinding for 60 min in N‐methylpyrrolidone (NMP) at a weight of 111^*^g. To eliminate the water layer between the layers of vermiculite, calcination was performed at 800 °C due to the robust forces between the layers. As a result, the vermiculite expanded and the spacing between the layers increased significantly (**Figure** **1**a, upper right). Using probe sonication, the arrangement of probes allowed the exfoliation of a single‐core layer. The FCL had a mean size and thickness of 5 nm (Figure 1a, bottom left).

Dispersibility and physiological stability are crucial parameters for nanomedicines intended for in vivo applications. To further modify the FCL, positively charged PEG‐NH_2_ was used, as shown in Figure 1a, bottom right, where the TEM image reveals the lamellar shape of the FCL. A bare FCL surface was positively charged (zeta potential 10.0 ± 0.7 eV), whereas a modified surface (zeta potential–17.0 ± 0.1 eV) indicated that PEG‐NH_2_ was successfully modified (Figure S1, Supporting Information). Scanning electron microscopy‐energy dispersive x‐ray (SEM‐EDX) elemental mapping revealed the presence of oxygen, nitrogen, and carbon in PEG‐NH_2_ and the FCL, which were also detected in FCL‐PEG (Figure 1b; Figure S2, Supporting Information), further confirming PEGylation. An evaluation of FCL and FCL‐PEG through DLS testing has been conducted, the results showing that the mean particle size of FCL and FCL‐PEG was 221.65 ± 0.24 nm and 230.13 ± 012 nm respectively (Figure S3, Supporting Information). The results revealed that the average particle size of FCL‐PEG increased by ≈9 nm compared to that of FCL. This increase in size is consistent with the expected outcomes of successful PEG coating, further confirming the modification. The successful functionalization of PEG‐NH_2_ was confirmed by the characteristic absorption peaks observed at ≈1200 and 2900 cm^−1^. These peaks are attributed to the stretching vibrations of ─C─O─C─ and ─CH in PEG‐NH_2_, respectively (Figure S4, Supporting Information).

After confirming the successful alteration of the FCL (Figure 1c), we assessed the photothermal conversion characteristics of FCL‐PEG. As shown in Figure S5 (Supporting Information), the FCL absorbs a broad range of light, from ultraviolet (UV) to near‐infrared (NIR). A TMB (3,3′,5,5′‐Tetramethylbenzidine) assay has been employed to further investigate the ability of FCL to generate reactive oxygen species (ROS) locally. The TMB assay is a sensitive method used to detect ROS production, and our results demonstrate a significant increase in oxidative activity within the infected microenvironment when exposed to FCL.^[^ ^23^ ^]^ These findings support our hypothesis that FCL reduces the antioxidant capacity of the microenvironment, thereby facilitating the accumulation of ROS which contributes to antimicrobial activity (Figure 1d). Figure 1e shows the swift and concentration‐dependent reduction in GSH, leading to the potential accumulation of ROS at the infection site. Previous studies have indicated that NIR light, specifically 808 nm laser light, achieves an optimal balance between tissue penetration, tissue self‐heating, and photothermal conversion. As a result, 808 nm lasers are the preferred choice for clinical photothermal therapy. To guarantee precision, the detection of the FCL photothermal conversion requires the use of an infrared radiation (IR) thermal camera and a conventional liquid thermometer. Figure 1f shows that when the FCL concentration was 200 ug mL^−1^ and power intensity was 2 W cm^−2^, the highest temperature increment (32.4 °C) was achieved. Figure 1g shows the rapid increase in temperature observed during the treatment of the FCL aqueous solutions using an 808 nm laser. Following the deactivation of the laser, the temperature of FCL‐ECMH gradually decreased to room temperature because of the temperature disparity. The photostability of FCL‐ECMH was demonstrated by the insignificant alterations observed in the photothermal conversion efficiency throughout the five heating and cooling cycles.

An ideal bioactive hydrogel must not only occupy the bone defect to offer the required mechanical assistance but also provide sufficient space and bioactive ions to stimulate the growth and restoration of new bone tissue. The hydrogel must undergo appropriate degradation and release ions to meet the requirements. To assess the biodegradability of FCL‐ECMH, the hydrogel was immersed in a Dulbecco's Modified Eagle Medium (DMEM) solution for three weeks, during which the weight loss was measured (Figure S6, Supporting Information). After three weeks of incubation, FCL‐ECMH exhibited a moderate rate of degradation, only 8% weight reduction. The Fe^3+^ and Mg^2+^ in FCL‐ECMH were slowly released as degradation proceeded (Figure 1h; Figure S7, Supporting Information). Therefore, FCL‐ECMH might possess excellent potential for facilitating the restoration of the affected tissue.

### 2.2 Antibacterial Property of FCL‐ECMH In Vitro

Previous research has indicated that iron overload combined with photothermal therapy can result in enhanced ferroptosis‐like death.^[^ ^24^ ^]^ First, explore FCL‐ECMH's bactericidal efficacy against the Gram‐positive bacterium Staphylococcus aureus. Six groups were respectively treated with PBS, FCL, FCL‐ECMH, NIR (2 W cm^−2^, 10 min), FCL + NIR (2 W cm^−2^, 10 min), and FCL‐ECMH + NIR (2 W cm^−2^, 10 min) to investigate the bactericidal activity of FCL‐ECMH.

**Figure 2:**
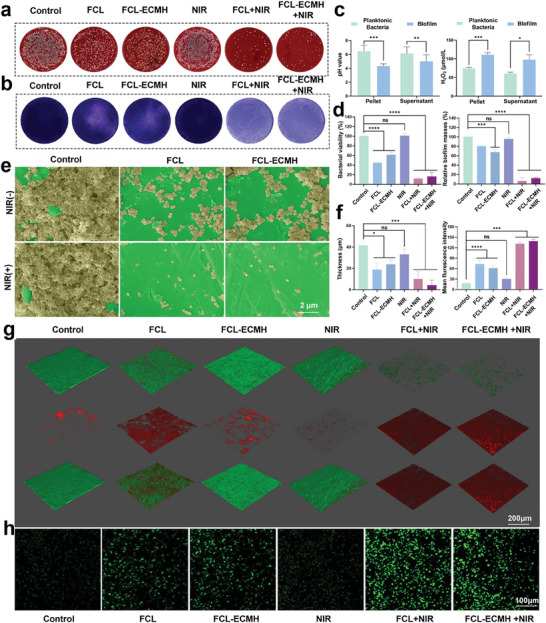
Antibacterial property of FCL‐ECMH in vitro. a) Colony‐forming units on blood agar plates of *S. aureus* after various treatments. b) The photographs of *S. aureus* biofilm were processed with different treatments and subjected to crystal violet staining. c) Results of pH value and H_2_O_2_ concentration in pellet and supernatant of planktonic *S. aureus* culture system. d) Bacterial viability and biofilm biomass of *S. aureus* biofilm. e) SEM images of *S.aureus* biofilms. Scale bar, 2 µm. f) Quantitative analysis of CLSM results. g) Confocal micrographs of the *S. aureus* biofilm in different groups. Dead bacteria (red), and live bacteria (green). Scale bar, 200 µm. h) The images of *S.aureus* biofilm staining with ROS probe in different groups. Scale bar, 100 µm. Data are presented as mean ± s.d, n = 3, ^*^ *p* < 0.1 ^**^ *p* < 0.01, ^***^ *p* < 0.001, ^****^ *p* < 0.0001, and ns = not significant.

To examine the antibacterial activity of FCL‐ECMH under laboratory conditions, the spread‐plate technique was used to quantify the number of surviving colonies (**Figure** **2**a). Subsequently, we assessed the inhibitory and eradication effects of FCL‐ECMH on biofilm formation using the crystal violet staining assay, as shown in Figure 2b. The residual biomass of biofilms was quantified by measuring the OD550 values. The results of the spread plate method and crystal violet staining are presented in Figure 2d, demonstrating a significant reduction in the number of surviving bacteria following treatment with the nanomedicine.

To determine the optimal conditions for promoting the interaction between FCL‐ECMH and the IME (Figure 2c), the chemical properties of the infection sites were analyzed. The biofilm aggregates in the *S. aureus* biofilm solution exhibited low pH and high concentration of H_2_O_2_. The IME offers an acidic setting and an ample supply of catalytic substrate for the nanozyme to produce toxic ·OH. This indicates that FCL‐ECMH has a good bactericidal effect at the site of infection. SEM results further corroborated the disruptive effects of FCL‐ECMH on biofilm structure. As depicted in Figure 2e, in comparison to the control group, bacterial cells exhibited a more sparse distribution following treatment with FCL‐ECMH. Additionally, confocal laser scanning microscopy (CLSM) was employed to view the residual bacteria and the 3D structure of the biofilm. The live/dead staining results, depicted in Figure 2g, illustrate live cells as green‐stained areas and dead cells as red‐stained areas. Comparatively, the control group exhibited a higher presence of green fluorescence signals compared to the FCL‐ECMH‐treated group. Through COMSTAT software, we analyzed the bio‐volume, average diffusion distance, and average thickness, all showing that the FCL‐ECMH + NIR group exhibits significant antibacterial effects(Figure S8, Supporting Information). The markedly lower bacterial survival rate in the FCL‐ECMH group compared to the control group may be due to the ROS catalytically generated by the Fenton reaction. The specific underlying mechanism is shown in Figure 2h where the FCL can utilize H_2_O_2_ in the IME to generate ROS that has a lethal effect on bacteria. The mechanism begins in the bacterial infection microenvironment, which typically contains trace amounts of hydrogen peroxide. The trivalent iron ions (Fe^3^⁺) in FCL‐ECMH react with this hydrogen peroxide to produce bivalent iron ions (Fe^2^⁺), water, and oxygen. The newly formed bivalent iron ions then undergo the Fenton reaction to produce ROS. *Escherichia coli* is another major pathogen responsible for postoperative infections. Investigating the bactericidal effect of FCL‐ECMH against it contributes to a comprehensive understanding of its antimicrobial properties. As depicted in Figures S9 and S10 (Supporting Information), FCL‐ECMH also demonstrates excellent bactericidal efficacy against *E. coli*.

### 2.3 In Vitro Effect of FCL‐ECMH on Macrophage Polarization

Macrophages function as immune cells and play a crucial role in maintaining immune balance and enhancing wound healing.^[^ ^25^ ^]^ Promoting polarization toward M2 is crucial in diabetic wound healing to restore immune homeostasis, making wound dressings of utmost significance. According to previous studies and our findings, the anti‐inflammatory effect of Fe^3+^ is likely to stem from its ability to regulate macrophage polarization and the immune microenvironment. Furthermore, it has been documented that Fe^3+^ facilitates polarization toward M2 to expedite wound healing. This study aimed to thoroughly examine the potential impact of FCL‐ECMH on polarization of M2 macrophages, both in a laboratory setting and in living organisms.

**Figure 3:**
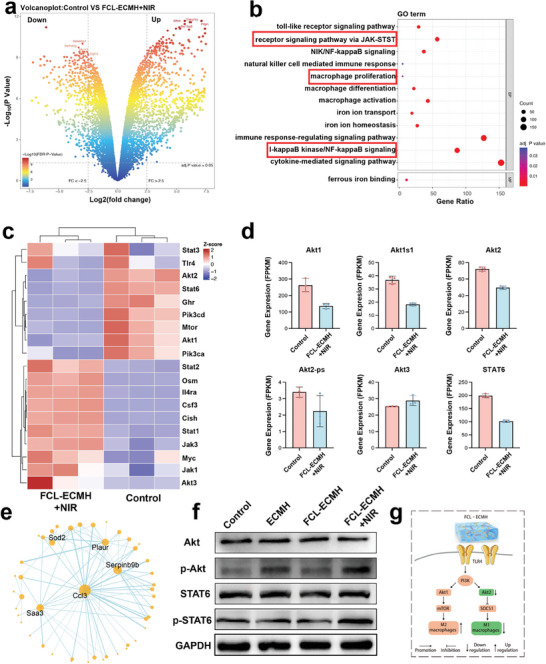
Morphological change, M2 phenotype polarization, and assessment molecular mechanisms using RNA‐seq of Raw 264.7 macrophages treated by the FCL‐ECMH. a) Volcano plot illustrating up‐regulated and down‐regulated gene expression between Control and FCL‐ECMH groups. b) GO enrichment analysis of up‐regulated genes in FCL‐ECMH groups. The bubble chart presents the enriched biological processes (BP) and molecular functions (MF) categories. Key pathways, such as the receptor signaling pathway via JAK‐STAT and macrophage proliferation are highlighted. c) Heatmap of JAK‐STAT pathway‐related gene expression between FCL‐ECMH and Control groups. Gene expression was standardized (z‐scores). d) Bar graphs depicting the expression levels of JAK‐STAT pathway‐related genes between Control and FCL‐ECMH groups. Gene expression is quantified in fragments per kilobase of transcript per million mapped reads (FPKM). e) Protein‐Protein Interaction (PPI) network generated from up‐regulated genes in the FCL‐ECMH group. f) Protein expression of STAT6, p‐STAT6, Akt, and p‐Akt were analyzed by western blot (n = 3). g) Schematic representation of the proposed mechanism by which FCL‐ECMH influences macrophage polarization.

To determine the possible molecular mechanisms underlying FCL‐ECMH‐induced macrophage polarization, RNA sequencing was employed to analyze changes in mRNA levels in macrophages co‐cultured with the control and FCL‐ECMH under FCL‐stimulated conditions for 24 h. The volcano plots shown in **Figure** **3**a demonstrated that 867 genes were expressed differentially (DEGs) between the FCL‐ECMH and the control group. Using the empirical Bayes method, 455 upregulated genes and 412 downregulated genes were identified. The analysis of the gene ontology (GO) pathway for the DEGs associated with the immune system revealed various immune defense mechanisms, including NF‐κB, Toll‐like receptor, JAK‐STAT, and TNF signaling pathway (Figure 3b). Notably, the JAK‐STAT6 pathway regulates M2 polarization. Figure 3c shows that the activation of the JAK‐STAT and PI3K/Akt signaling pathways was significantly induced by upstream cytokines, hormones, and membrane receptors, according to the enrichment analysis of the KEGG signaling pathway. In this study, western blotting was used to confirm the signaling pathways involved in FCL‐ECMH‐induced macrophage polarization. As shown in Figure 3f, after treatment with FCL‐ECMH, there was a minimal increase in the expression of Akt, STAT6, and p‐STAT6 protein, suggesting that the JAK‐STAT6 pathway was active compared to that in the control.

Figure 3d,e shows that the PI3K/Akt signaling pathway plays a role in the regulation of macrophage polarization by FCL‐ECMH, which is activated by Toll‐like receptor 4 (TLR4). Among the members of the Akt family, *Akt2* is involved in M1 macrophage polarization, while *Akt1* and *Akt3* are involved in M2 macrophage polarization. FCL‐ECMH significantly increased expression of the *Akt1* and *Akt3* gene and *Akt2* expression significantly decreased (*p* < 0.05). Additionally, the FCL‐ECMH group exhibited a significant upregulation of mTOR gene expression, a crucial transcription factor targeted by *Akt1*, compared to the other groups (*p* < 0.05). Furthermore, *Akt2*‐induced M1 polarization is associated with enhanced *RelA/NF‐κB* activation, which is regulated by the suppression of the cytokine signaling inhibitor (SOCS1). The findings of this study demonstrated that FCL‐ECMH had the ability to efficiently trigger M2 macrophage polarization via the PI3K/Akt1/mTOR signaling pathway (Figure 3g).

### 2.4 In Vitro, FCL‐ECMH Modulates the Polarization of Macrophages

Tissue healing is essential for the comprehensive treatment of infections. Moreover, thermal therapy may potentially lead to damage in soft tissues, thereby affecting wound healing.^[^ ^26^ ^]^ In recent years, a considerable amount of research has focused on achieving anti‐infection or pro‐healing purposes through the phenotypic switching of macrophages. This study aims to promote macrophage M2 polarization through the immunomodulatory ability of FCL‐ECMH, thereby stimulating wound healing.

**Figure 4:**
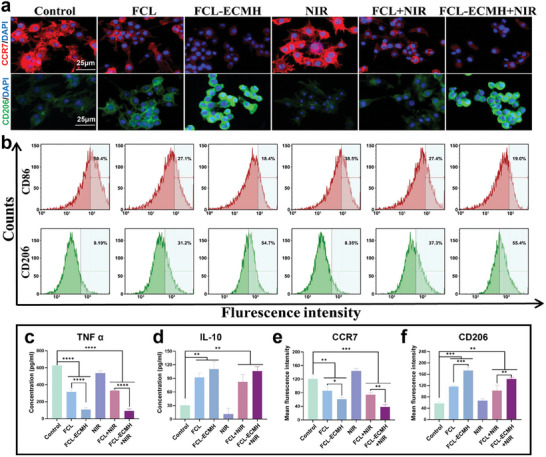
FCL‐ECMH regulated macrophage cells. a) Immunofluorescent staining was performed on RAW264.7 macrophages, highlighting CCR7 expression (red, M1 marker), CD206 expression (green, M2 marker), and nuclei stained with DAPI (blue). Scale bar, 25 µm. b) The expression levels of CD86 (M1) and CD206 (M2) in macrophages were analyzed using flow cytometry. c,d) ELISA results of proinflammatory and anti‐inflammatory cytokines (TNFα and IL‐10). e,f) Quantitative analysis of immunofluorescent results. Data are presented as mean ± s.d, n = 3, ^*^ *p* < 0.1, ^**^ *p* < 0.01, ^***^ *p* < 0.001, and ^****^ *p* < 0.0001.

We selected RAW264.7 cells as the target to verify the immunomodulatory effects of FCL‐ECMH. As shown in **Figure** **4**a, immunofluorescence staining was employed to verify the demonstrate that potential of the hydrogels, and increased expression of CD206 (M2 marker, indicated by green fluorescence), and decreased the expression of CCR7 (M1 marker, indicated by red fluorescence) was observed, suggesting a shift toward anti‐inflammatory M2 polarization. Figure 4e,f further supports these findings with quantitative analysis of the mean fluorescence intensities. These results were in accordance with our flow cytometry result. The proportion of M2 cells labeled with CD206 increased significantly to a minimum of 55.4%, compared to only 8.35% in the NIR group, highlighting a substantial difference in immunomodulatory effects (Figure 4b). Additionally, enzyme‐linked immunosorbent assay (ELISA) results, depicted in Figure 4c,d, showed a reduction in pro‐inflammatory cytokines like TNF‐α and an increase in anti‐inflammatory IL‐10 levels. These comprehensive findings lead us to conclude that FCL‐ECMH effectively stimulates anti‐inflammatory M2 polarization in the repair period, showcasing its potential to enhance wound healing by immune modulation.

### 2.5 FCL‐ECMH Promoted Osteogenesis and Angiogenesis In Vitro

**Figure 5:**
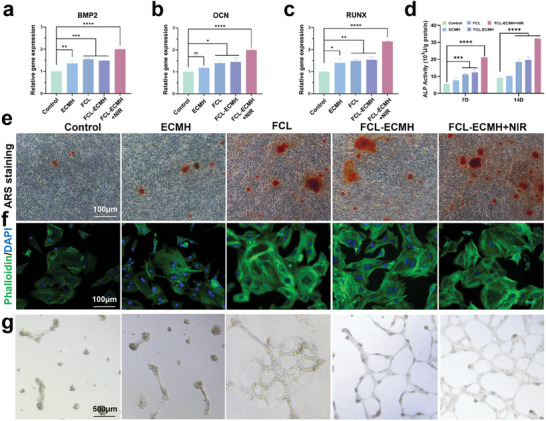
Evaluation of promoting tissue repair function in vitro a–c) The expression of related osteogenic genes (*BMP2*, *OCN*, and *RUNX2)* was detected by RT‐qPCR. d) The results of ALP activity were detected by co‐culture for 7 and 14 days. e) ARS of different samples after 14 days, Scale bar, 100 µm. f) Fluorescence images were obtained of BMSCs co‐stained with phalloidin and DAPI. Scale bar, 100 µm. g) Representative images of tubular formation of HUVECs after 24 h incubation. Scale bar, 500 µm. Data are presented as mean ± s.d, n = 3, ^*^ *p* < 0.1 ^**^ *p* < 0.01, ^***^ *p* < 0.001, ^****^ *p* < 0.0001, and ns = not significant.

To assess the tissue repair capabilities of FCL‐ECMH, we conducted in vitro studies focusing on their osteogenic and vasogenic abilities. The osteogenic potential was first evaluated by co‐culturing bone marrow mesenchymal cells (BMSCs) with FCL‐ECMH, as shown in **Figure** **5**a–c. Osteogenic protein expression, including *BMP2, OCN, and RUNX2*, was quantified using RT‐qPCR assays to determine the hydrogel's effectiveness in enhancing osteogenesis. Notably, the FCL‐ECMH+NIR group demonstrated significantly higher osteogenic activity than the other groups. Further osteogenic differentiation was assessed by measuring alkaline phosphatase (ALP) activity across different treatments, with results that corroborated the PCR findings, as shown in Figure 5d. Additionally, extracellular osteogenic mineralization was analyzed using alizarin red staining (ARS), revealing increased mineral deposition in both the FCL‐ECMH and FCL‐ECMH + NIR groups, illustrated in Figure 5e. We then used fluorescence confocal microscopy to observe the differentiation of bone marrow mesenchymal stem cells under the intervention of the material. The results revealed a significantly enlarged cytoskeleton in the FCL‐ECMH + NIR group, indicating enhanced osteogenic activity. The FCL‐ECMH + NIR group displayed notably wider BMSCs, as depicted in Figure 5f. Furthermore, the angiogenic potential of ECMH enhanced by the nutritional effects of Mg was explored through a tube formation assay. This assay showed an increased number of junctions and branches, key indicators of vascular formation, in the FCL‐ECMH group (Figure 5g).

### 2.6 In Vivo, FCL‐ECMH+NIR Effectively Treats Diabetic Wounds

**Figure 6:**
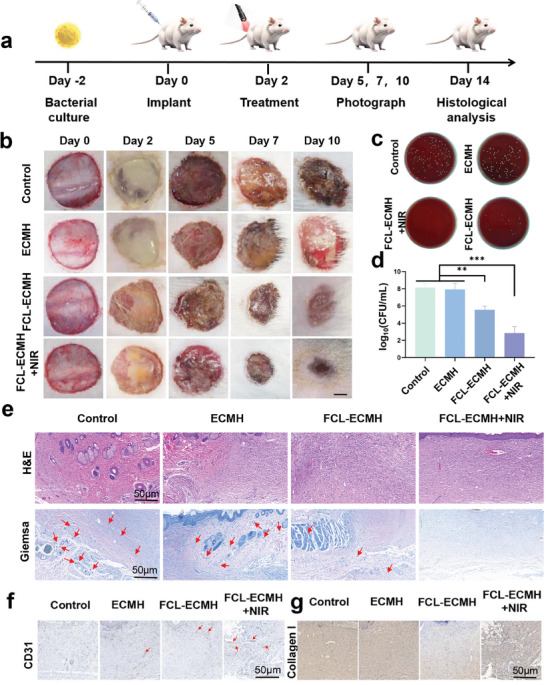
FCL‐ECMH eliminates diabetic wound infections in vivo. a) Schematic of the diabetic wound infections model and experimental procedures. b) Macroscopic photos of the diabetic wound in various treatment groups. Scale bar: 0.2 cm. c) Bacterial colonies cultured from wound tissues. d) CFU counts of wound tissue homogenates via SPM. e) Representative H&E staining of wound tissues at various instants in multiple groups. Scale bar: 50 µm. Representative Giemsa staining of wound tissues at various instants across all groups. Scale bar: 50 µm. f,g) Representative immunohistochemical staining of CD31 and collagen type I of wound tissues section at various instants. Scale bar: 50 µm. Data are presented as mean ± s.d, n = 6, ^**^ *p* < 0.01 and ^***^ *p* < 0.001.

Encouraged by the promising in vitro outcomes of FCL‐ECMH, we investigated its anti‐biofilm effectiveness using an in vivo model simulating diabetic wound infections.^[^ ^27^ ^]^ The comprehensive procedure is presented in **Figure** **6**a. The successful construction of the diabetic wound infections model was evidenced by the prominent exudation and pus moss formation in all four groups on day 2. On days 2, 5, 7, and 10, we obtained comprehensive visual records of the rats to evaluate both the infection progression and skin status (Figure 6b). The temperature at the wound site increased to 55 °C within ≈5 min of NIR exposure. Surprisingly, significant skin inflammation and swelling were observed in both the control and FCL groups. In contrast, few signs of infection or skin lesions were visible in the FCL + NIR and FCL‐ECMH + NIR groups. Bacterial infection severity was quantified through the enumeration of colony‐forming units within the infected tissue, as illustrated in Figure 6c,d. The FCL + NIR and FCL‐ECMH + NIR groups exhibited a ≈6‐log decrease in colony numbers, indicating a substantial reduction in bacterial load. Histopathological examination of the local skin tissues (Figure 6e) involved Hematoxylin Eosin and Giemsa staining. H&E staining revealed a notable reduction in subcutaneous connective tissue thickness, serving as an indicator of inflammation and edema severity in the FCL + NIR and FCL‐ECMH + NIR groups. As shown in Figure 6g, the control and FCL groups showed a substantial bacterial presence, in contrast to the FCL + NIR and FCL‐ECMH + NIR groups, which displayed minimal pathogens, as indicated by the red arrows. Subsequently, we investigated the anti‐inflammatory potential of the FCL and FCL‐ECMH in vivo. Blood vessel formation and tissue regeneration in the entire wound bed were evaluated using immunohistochemistry (IHC) for CD31 and type I collagen. The strong expression of CD31 and type I collagen observed in the subcutaneous tissue of the NIR groups demonstrated the excellent effectiveness of the FCL‐ECMH nanoplatform (Figure 6f,g). ImageJ software was used to quantitatively assess the wound closure rate in different groups (Figure S11, Supporting Information). The results showed that the FCL‐ECMH+NIR treatment group significantly reduced the wound area on day 10. Additionally, we have conducted a quantitative assessment of the histological data by counting CD31‐positive cells involved in angiogenesis, which are crucial for wound healing(Figure S12, Supporting Information). The results showed that the FCL‐ECMH+NIR treatment group significantly increased the proportion of CD31 positive cells. In summary, these findings confirmed the anti‐biofilm and regenerative effects of FCL‐ECMH, even within an intricate in vivo milieu.

### 2.7 FCL‐ECMH Promoted Osteogenic In Vivo

**Figure 7:**
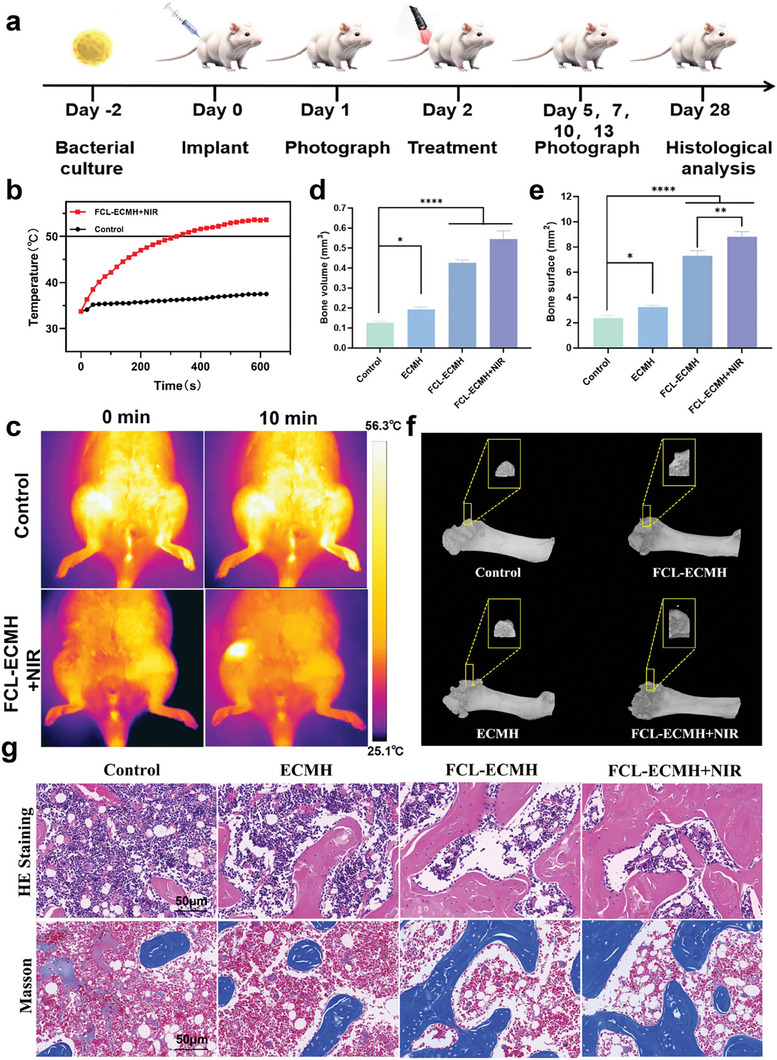
Application of FCL‐ECMH to in vivo model. a) Schematic illustration of in vivo osteogenic property evaluations. b) The temperature‐increase curves. c) Infrared images of treatment sites before and after being irradiated. d,e) The quantification of bone volume and bone surface. f) The 3D pictures of different groups were measured by Micro‐CT. g) H&E staining and Masson's staining images. Scale bar, 50 µm. Data are presented as mean ± s.d, n = 6, ^*^ *p* < 0.05, ^**^ *p* < 0.01 and ^****^ *p* < 0.0001.

The osteogenic efficacy of FCL‐ECMH was investigated in a bacterial infection bone defect model. The overall protocols and time intervals of the animal experiments are depicted in **Figure** **7**a. On Days 0 and 1, a circular defect was surgically generated in the femoral condyle. Subsequently, bone infection was induced by injecting 10^7^ colony‐forming units (CFU) of *S. aureus*. General photographs were taken to systematically record the overall physiological status of the rats. On day 2, the infection sites were subjected to a 10‐min NIR exposure, and alterations in temperature were meticulously documented using an IR camera (Figure 7b,c). The temperature at the treatment sites in the FCL‐ECMH groups increased rapidly, stabilizing at ≈50 °C for 5 min. In contrast, the control groups experienced a minimal increase in temperature, stabilizing between 35 and 40 °C.

The in vitro osteogenic capabilities of FCL‐ECMH warrant further exploration through in vivo investigations. The results of microcomputed tomography indicated that the volume of newly regenerated bone in the FCL‐ECMH+NIR group significantly exceeded that of the control group (Figure 7f). Quantitative analysis of the bone volume and surface area further underscored the significance of this effect, demonstrating a substantial increase in the FCL‐ECMH group (Figure 7d,e). The severity of local inflammation and the extent of bone regeneration were assessed using H&E and Masson's stains(Figure 7g; Figure S13, Supporting Information). Both analyses consistently illustrated significant regeneration of the femoral condyle. Moreover, the control group exhibited significant infiltration of inflammatory cells as revealed by H&E staining, contrasting with a noticeable decrease noted in the FCL‐ECMH group. At the same time, we tested the hydrogel toxicity by CCK‐8 assay, and the results showed good cytocompatibility (Figure S14, Supporting Information). H&E staining was performed on major rat organs, including cardiac, hepatic, splenic, pulmonary, and renal tissues (Figure S15, Supporting Information). These outcomes indicate the absence of significant damage, underscoring the superior biocompatibility of FCL‐ECMH.

To explain these findings, we have identified two primary mechanisms responsible for the enhanced osteogenic properties: 1) localized sterilization: NIR radiation induces localized heating of the FCL‐ECMH hydrogel. This thermal effect not only eradicates pathogenic bacteria at the infection site but also reduces overall infection levels, creating a more favorable environment for bone regeneration. 2) ion release and pathway activation: The NIR irradiation further facilitates the release of bioactive ions such as iron and magnesium from the hydrogel. The increased availability of these ions activates the JAK‐STAT signaling pathway, pivotal for promoting the differentiation of bone marrow mesenchymal stem cells into osteoblasts, essential for new bone formation.

## 3 Conclusion

In this study, we effectively formulated FCL‐ECMH using natural polymers, endowing it with excellent immunomodulatory properties. This innovative tissue filler not only combats bacterial invasion in the very early stages but also facilitates an anti‐inflammatory response and tissue restoration in the later stages, harmonizing with the biodegradation of the hydrogel. The dual nature of its chemical and physical cross‐linking affords FCL‐ECMH robust mechanical integrity while ensuring commendable cytocompatibility and formidable antibacterial function.

Our findings highlight the active involvement of FCL‐ECMH in promoting M2 macrophage polarization, which is orchestrated via the JAK‐STAT signaling cascade. In our animal models of tissue infection, the early implantation phase demonstrated the ability of FCL‐ECMH to eliminate bacteria and inhibit inflammation. As the study progressed, the hydrogel became instrumental in steering macrophages toward the M2 phenotype, laying the foundation for enhanced angiogenesis and osteogenesis. Our in vivo evaluations further confirmed the ability of FCL‐ECMH to accelerate infected wound healing by promoting M2 polarization, dampening inflammation, and fostering angiogenesis.

FCL‐ECMH has shown promise as a tissue filler, noted for its straightforward production, economic viability, and logistical ease in transportation and storage. Its efficacy in clinical settings suggests strong potential for managing orthopedic complications and use in emergency medicine. Additionally, studies in larger animal models and eventual clinical trials are necessary to confirm these findings and expand the applicability of FCL‐ECMH in clinical practice.

## 4 Experimental Section

### Preparation of FCL‐ECMH

FCL was prepared by grinding, calcining, alkaline etching, and sonication.^[^ ^28^ ^]^ First, vermiculite powder (1 g) was dispersed in an NMP solution (50 mL), and the solution was ground for 30 min. The ground vermiculite powder was calcined at 800 °C for 2 h, in a furnace, while exposed to an atmosphere of nitrogen gas after undergoing washing and drying. The heated vermiculite powder was placed in a sealed autoclave containing a saturated NaOH solution (100 mL). The airtight autoclave was positioned inside a preheated furnace at 150 °C for 24 h. The NaOH‐treated vermiculite solution was subjected to centrifugation and three rounds of washing to remove the remaining NaOH. The mixture was ultrasonicated for 5 h in an NMP solution. The sonicated liquid was centrifuged at 16099 times gravity and rinsed three times with deionized water. Subsequently, a solution of PEG‐NH_2_ (10 mg) was added to the FCL solution (5 mL, 0.1 mg mL^−1^) and agitated for 12 h after 30 min of sonication. The Porcine acellular dermal matrix fragments were solubilized in 0.01 mol L^−1^ hydrochloric acid (HCl) and subjected to continuous stirring and enzymatic digestion with pepsin at ambient temperature until they were essentially dissolved. Following digestion, the pH was adjusted to neutral, and the solution was subsequently diluted with 1 × phosphate‐buffered saline (PBS, pH 7.4) to a final concentration of 10 g L^−1^ to prepare the pre‐gel solution. This pre‐gel was then incubated at 37 °C for 30 min to facilitate hydrogel formation. Employing a similar methodology, acellular dermal matrix hydrogels of various mass concentrations were synthesized. The FCL (0.1 mg mL^−1^) was dissolved in a hydrogel solution of extracellular matrix, at a concentration of 2% (w v^−1^), and maintained at a temperature of 45 °C for 1 h. ECMH crosslinked was successfully obtained with FCL using this method.

### Physicochemical Characterization of FCL‐ECMH

The freeze‐dried FCL‐ECMH and FCL were examined using an SEM (Gemini 300, ZEISS, Germany) and a TEM (Tecnai G2 F30, FEI, Netherlands). The DLS size and ZETA potential were determined using a Zetasizer Nanoseries (Malvern, UK). A physicochemical characterization of FCL‐ECMH was conducted. The UV spectra were acquired using a UV‐2700i spectrophotometer (SHIMADZU, Kyoto, Japan).

### Photothermal Performance of FCL‐ECMH

The photothermal efficiency of FCL‐ECMH was tested by subjecting it to an 808 nm laser with varying power densities (0.5 to 2 W cm^−2^) for 5 min, using concentrations ranging from 0.05 to 0.2 mg mL^−1^. An IR thermal camera was used to detect the increase in temperature of the FCL‐PEG solution.

### TMB Oxidation Experiment

The peroxidase‐like activity of the FCL nanocomposites was evaluated by assessing their ability to oxidize TMB, a chromogenic colorless substance, into its blue‐colored intermediate charge transfer complex, oxTMB. This oxidation was monitored by measuring the UV–vis absorbance of oxTMB at its maximum wavelength of 652 nm. For this assay, 1.0 mM TMB and varying concentrations of FCL (0, 100, 200, and 400 µg mL^−1^) were added to a PBS buffer solution (pH 7.4). After incubating for 30 min, the absorbance at 652 nm was recorded to evaluate the ROS‐producing capability of the nanocomposites.

### Spread Plate Method

In this research, Staphylococcus aureus (S. aureus, ATCC 43300) was employed. The spread plate method was utilized for enumerating bacterial colonies. Following various treatments, the biofilm was suspended in PBS and diluted to a concentration of 1 × 10^4^‐fold. Subsequently, 100 µL of the diluted PBS solution was spread onto blood agar plates. Following incubation at 37 °C for 24 h, the numbers of bacterial colonies were counted.

### Crystal Violet Staining

The crystal violet staining was utilized to determine the biofilm biomass. After being washed with PBS three times, the biofilms were stained with a crystal violet staining solution. Following a 20‐min incubation period, the crystal violet solution was discarded, and the biofilm was washed with PBS. Finally, the biofilm was resolubilized with 30% glacial acetic acid, and the absorbance of the eluate at 590 nm was measured.

### Scanning Electron Microscopy

Biofilm grown on titanium sheets was exposed to different treatments. Afterward, the biofilm was fixed with electron microscopy fixation solution at 4 °C, overnight. Different ethanol gradients (30%, 50%, 70%, 80%, 95%, and 100%) were used to dehydrate the biofilm for 10 min at each concentration. After lyophilization and gold spraying, the biofilm formation was observed with SEM.

### Bacterial Live/Dead Staining

The biofilm was cultivated within confocal dishes to investigate its 3D structure. After different treatments, it was rinsed and subsequently stained using the LIVE/DEAD BacLight Bacterial Viability Kit according to the manufacturer's instructions. The structure of the biofilm was observed with a CLSM (ZEISS LSM 710, Germany). Additionally, biomass quantification was conducted utilizing the COMSTAT plugin in ImageJ software. CLSM‐captured images, arranged as z‐stacks through 3D reconstruction, comprised a series of images with 1‐µm intervals in the z‐section from the substrate (disk) to the top of the biofilm. Subsequently, three parameters from COMSTAT were chosen to quantify biofilm structures: bio‐volume, average diffusion distance, and average thickness.

### Macrophage Polarization

Detection of M1 and M2 macrophage polarization markers used immunofluorescence staining, RT‐qPCR, and western blotting. Initially, Raw 264.7 cells were distributed at a concentration of 2 × 10^5^ cells per well onto a six well plate and incubated overnight. Cells were incubated in DMEM with LPS (1 µg mL^−1^) for 12 h. Then, cells were incubated in DMEM containing FCL‐ECMH for 24 h. For immuno‐fluorescent staining, cells were stained with CD206 overnight at 4 °C. CLSM (Olympus, Tokyo, Japan) was used to obtain images.

To perform RT‐qPCR, cells were subjected to isolation and extraction of total RNA using the FastPure cell/tissue total RNA isolation kit (Vazyme, RC101‐01, China). RT‐qPCR was used to measure CD86, TNF‐α, and CD206 expression. ChamQ Universal SYBR qPCR Master Mix (Q711; Vazyme, China) was used for quantification. Analysis was performed using the 2^−ΔΔCt^ approach. GAPDH was used to normalize the expression of all genes.

### Osteogenesis and Angiogenesis Assays (In Vitro)

Real‐time PCR method was applied to assess the osteoblastic differentiation of BMSCs. Osteogenesis related genes including *BMP2*, *RUNX2*, and *OCN* were assessed. After culturing the cells for 14 days, the cells were collected to extract the total RNA using the RNA Purification Kit (EZBionscience). A Color Reverse Transcription Kit (EZBionscience) was used to convert RNA into cDNA through reverse transcription. The QuantStudio 7 Flex system (Life Technologies, Carlsbad, CA, United States) was used to conduct RT‐qPCR with cDNA using the 2 × Color SYBR Green qPCR Master Mix (EZBionscience). The primer sequences are shown in Table S1 (Supporting Information).

To measure ALP activity in cells after culturing for 7 and 14 days, an ALP assay kit (ab83369, Abcam, Cambridge, UK) was used according to the manufacturer's protocol. A bicinchoninic acid (BCA) protein assay kit (Solarbio, China) was used to detect the protein content. For ARS staining, BMSCs were fixed with 4% paraformaldehyde after culturing for 14 days and then stained for 30 min with Alizarin Red. After rinsing the samples with PBS, they were examined and photographed with an optical microscope.

The angiogenic potential of FCL‐ECMH was assessed through a tube formation assay. For this assay, 24‐well plates were uniformly coated with Matrigel (BD, Franklin Lakes, NJ, USA). After 6 h, cells were photographed with a light microscope, and the number of nodes, meshes, and junctions were analyzed by Image J.

### Diabetic Wound Healing Experiments

All animal experiments were approved by the Animal Welfare Ethics Committee of Shanghai Tenth People's Hospital (SHDSYY‐2022‐4443). Male Sprague‐Dawley (SD) rats were intraperitoneally administered streptozotocin (STZ, Macklin, China) for three consecutive days to conduct experiments on healing diabetic wounds. After 21 days, diabetic rat models were established by consistently surpassing glucose levels of 16.7 mmol L^−1^. Biopsy punches were used to create full‐thickness wounds measuring 6 millimeters in diameter on the dorsal sides of each rat, and 10^7^ CFU of *S. aureus* were added into the wounds. FCL‐ECMH was sequentially applied to the wounds. A dressing is used to cover the wound throughout the procedure. Pictures of the wound areas were taken on days 0, 2, 5, 7, and 10 days. The treatment involved 808 nm laser irradiation for 5 min on days 1 and 3 following the initial hydrogel implantation, totaling two sessions.

### Bacterial Infected Bone Defect Model Construction

All animal experiments were approved by the Animal Welfare Ethics Committee of Shanghai Tenth People's Hospital (SHDSYY‐2022‐4443). Construction of a model for bone defects infected with bacteria involved randomly dividing 20 SD rats, aged 9 weeks, into four groups: control, ECMH, FCL‐ECMH, and FCL‐ECMH+NIR. A bone infection model was established by creating a defect with a drill, measuring 0.6 mm in diameter, in the femoral condyle, followed by the introduction of 10^7^ CFU of *S. aureus*. Various nanoparticles were administered, followed by laser treatment (2 W cm^−2^, 10 min) on the subsequent day. After 4 weeks, all rats were sacrificed and regenerated bone was analyzed with microcomputed tomography images. After preparing the histological samples by paraffin sections, Giemsa, Masson, and immunofluorescence staining were conducted.

### Bacterial Colony Counting

For standard plate counting tests, the surrounding wound tissue (1 g) was collected and diluted 100 times using phosphate buffered saline (PBS). Subsequently, 100 µL of the diluted PBS solution was spread onto blood agar plates. Following incubation at 37 °C for 24 h, the numbers of bacterial colonies were counted.

### Statistical Analysis

The in vitro experiments were performed at least three times, and the in vivo experiments were conducted a minimum of six times. Data are presented as the mean ± standard deviation. Statistical analyses were carried out using GraphPad Prism software (version 9.0) or Origin (Version 2019b). We employed Student's t‐test and one‐way analysis of variance (ANOVA) to determine statistical significance, which was set at P<0.05. The significance levels are indicated as follows: ^*^ *p* < 0.05, ^**^ *p* < 0.01, ^***^ *p* < 0.001, ^****^ *p* < 0.0001; ns denotes no statistical significance.

## Conflict of Interest

The authors declare no conflict of interest.

## Supporting information

Supporting Information

## Data Availability

The data that support the findings of this study are available from the corresponding author upon reasonable request.
