# Supplementary material for: A Multifunctional Tissue‐Engineering Hydrogel Aimed to Regulate Bacterial Ferroptosis‐Like Death and Overcoming Infection Toward Bone Remodeling
Source: Adv Sci (Weinh). 2024 Jun 19;11(30):2309820. doi: 10.1002/advs.202309820 (PMC11321691; doi:10.1002/advs.202309820)
Supplement: Supplementary file 1 — Supporting Information [file ADVS-11-2309820-s001.docx]

Supporting Information

**A Multifunctional Tissue-Engineering Hydrogel Aimed to Regulate Bacterial Ferroptosis-like Death and Overcoming Infection toward Bone Remodeling**

*Renjie Lu^1^, Zhiyuan Luo^1^, Yuanyuan Zhang, Jiahao Chen, Yang Zhang*, Chi Zhang**

**1. Supplementary Methods**

**1.1 Materials.**

Vermiculite, N-methylpyrrolidone, PEG-NH_2,_ was purchased from Sinopharm Chemical Reagents Co., Ltd.. Alexa Fluor 488-labeled anti-CD80 antibody, APC-labeled anti-CD86 antibody and PE-labeled anti-CD206 antibody were purchased from BioLegend (USA). The RNA Purification Kit, Color Reverse Transcription Kit, 2×Color SYBR Green qPCR Master Mix were purchased from EZBionscience. ELISA kits (TNF-α, IL-10) were purchased from Multi Science Biotech Co. (China).

**1.2 In vitro cytotoxicity.**

Diverse concentrations of FCL (0, 50, 100, 200, 400, 800 μg mL^-1^) were used to incubate with HUVEC, RAW264.7, and BMSCs for 24 h to determine the cell viability using a cell counting kit 8 (CCK-8). We seeded the cells at a density of 8000 cells per well in 96-well plates and incubated them at 37℃ in an atmosphere of 5% CO_2_. After 24 h of culture, CCK-8 and medium were mixed at a ratio of 1 : 10 to add to the wells for incubation for 2 h. The absorbance at 450nm was then measured using a microplate reader (Epoch BioTEK, USA) after an additional 1.5 hours of incubation.

**1.3 Bacterial strains and biofilm culture.**

In this research, Staphylococcus aureus (*S. aureus*, ATCC 43300) was employed. Planktonic bacteria were cultured in tryptic soy broth (TSB) medium at 37 ℃ with shaking (250 rpm) overnight for subsequent experiments. Titanium discs were incubated overnight at 37°C with 10^6^ CFU suspension of bacteria for biofilm cultivation. In the following experiments, the bacterial suspension was discarded.

**1.4 Characterization.**

Images and EDS were acquired on JEM-2100F operating at 200 kV for transmission electron microscopy, HRTEM, and electron dispersive spectroscopy (EDS). Confocal laser scanning microscopy (CLSM) images were taken with Olympus FV1000, a confocal microscope. SEM images were taken with Hitachi S-4800.

1. **Supplementary Tables and Figures**

**Table S1.** Primers sequences used in PCR experiment.^[1]^

| **Gene** | **Upper primer sequence (5’ to 3’)** | **Lower primer sequence (5’ to 3’)** |
| --- | --- | --- |
| GAPDH | CATGTTCCAGTATGACTCCACTC | GGCCTCACCCCATTTGATGT |
| OCN | CTGACCTCACAGATCCCAAGC | TGGTCTGATAGCTCGTCACAAG |
| Runx2 | TCGGAGAGGTACCAGATGGG | AGGTGAAACTCTTGCCTCGT |
| BMP2 | TTTAGGGTTAGGAGAGCGAGG | GACGATCTCGATACCAAACG |


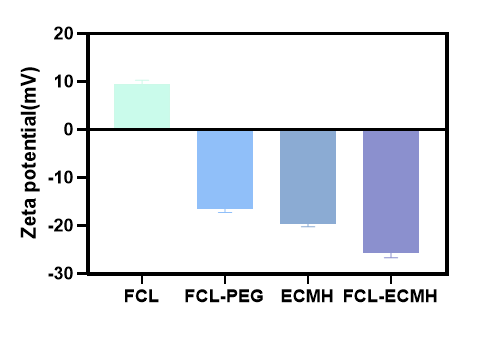


**Figure S1.** Zeta potential of FCL, FCL-PEG, ECMH, FCL-ECMH.


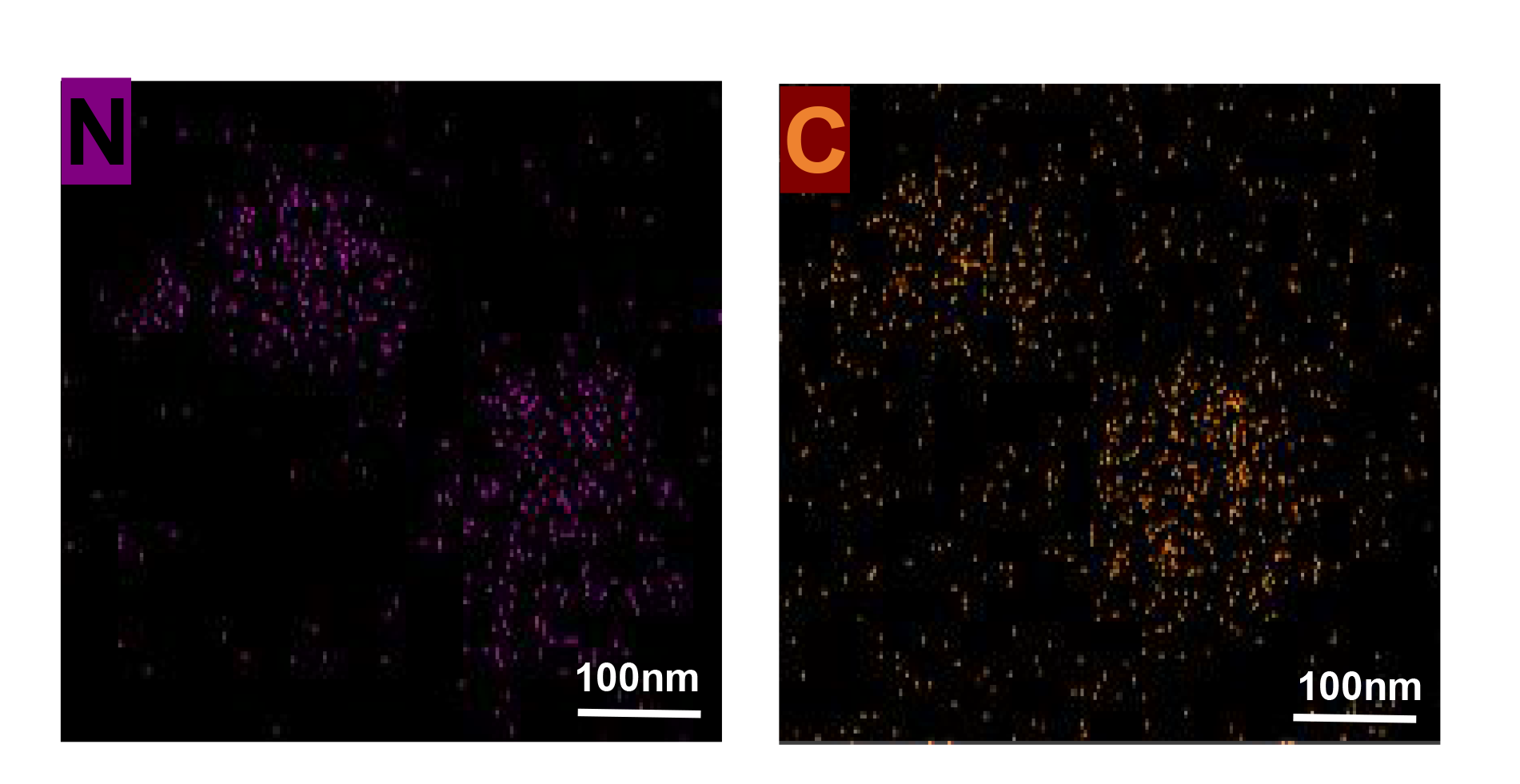


**Figure S2.** SEM-EDX mapping images of FCL (scale bar 100 nm for all panels).

***
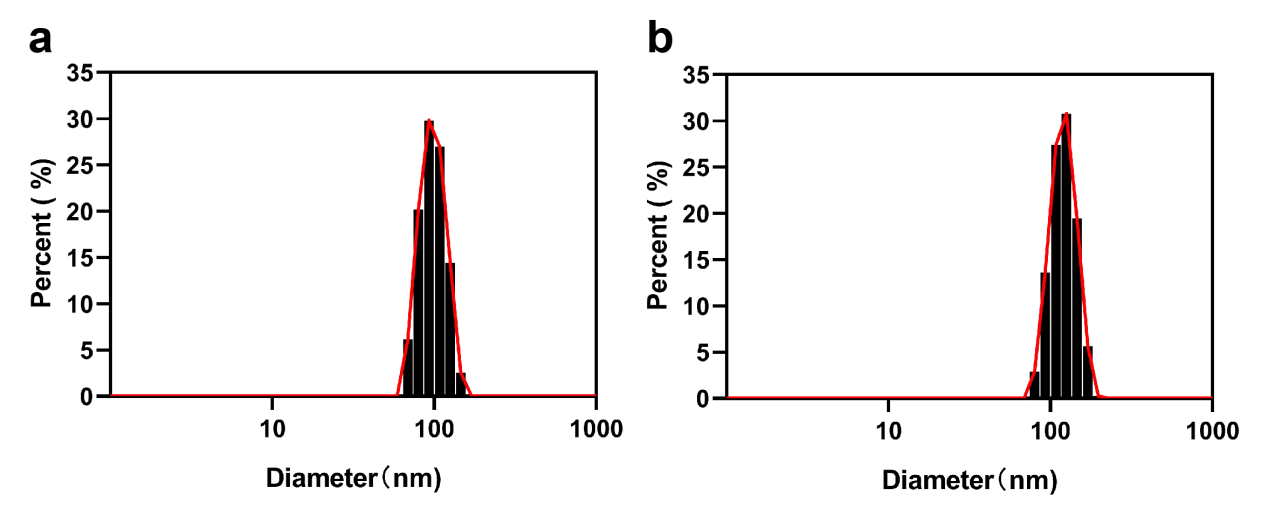
***

**Figure S3.** Particle size distribution of (a) FCL and (b) FCL-PEG.


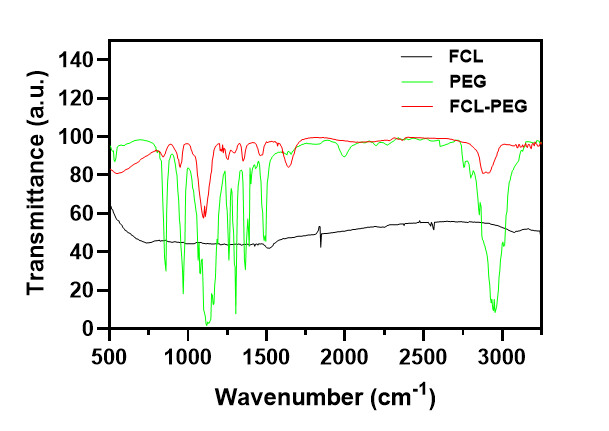


**Figure S4.** FTIR spectrum of FCL, PEG-NH_2_, and FCL-PEG.

**Figure S5.** The UV–vis absorption spectra of FCL with different concentrations.


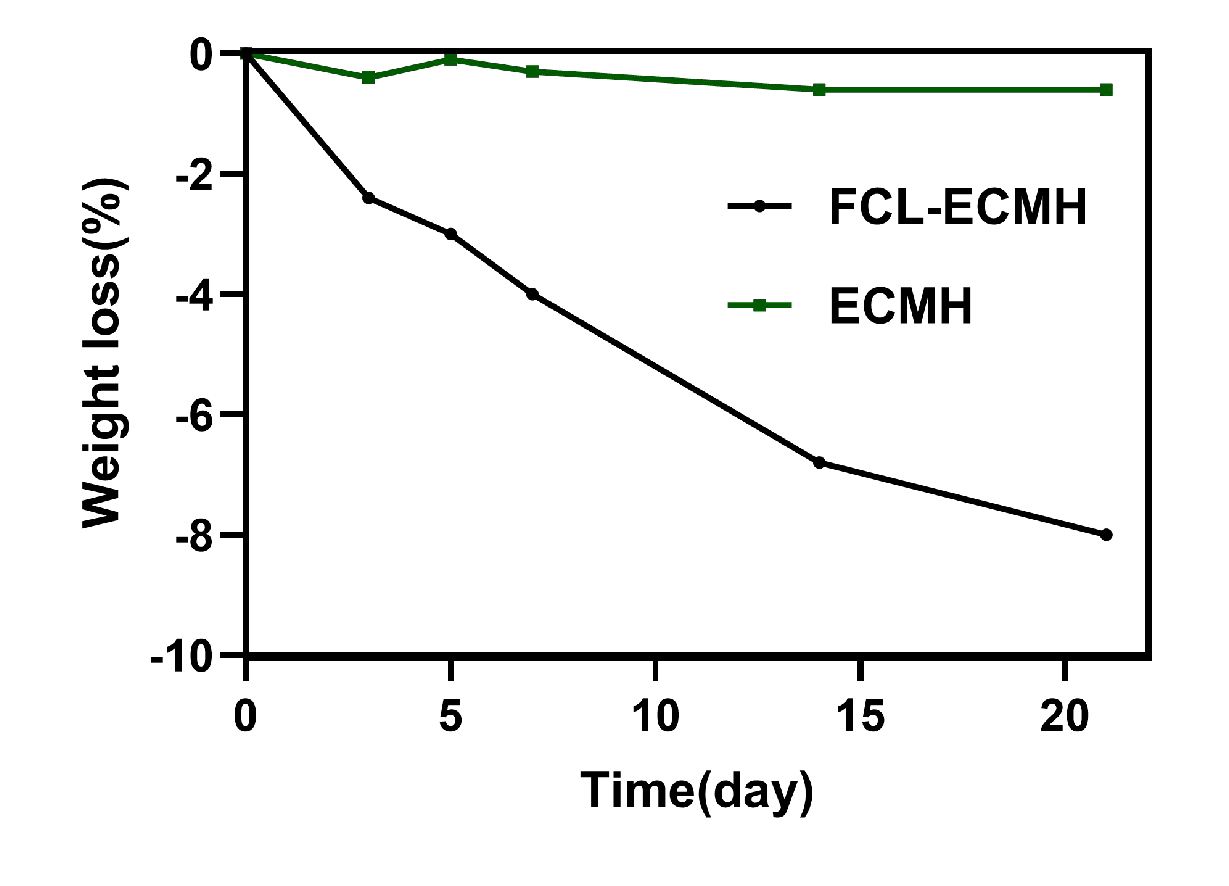


**Figure S6.** It took FCL-ECMH 28 days to degrade and lose approximately 8 weight percent (mean ± SD, n = 3)


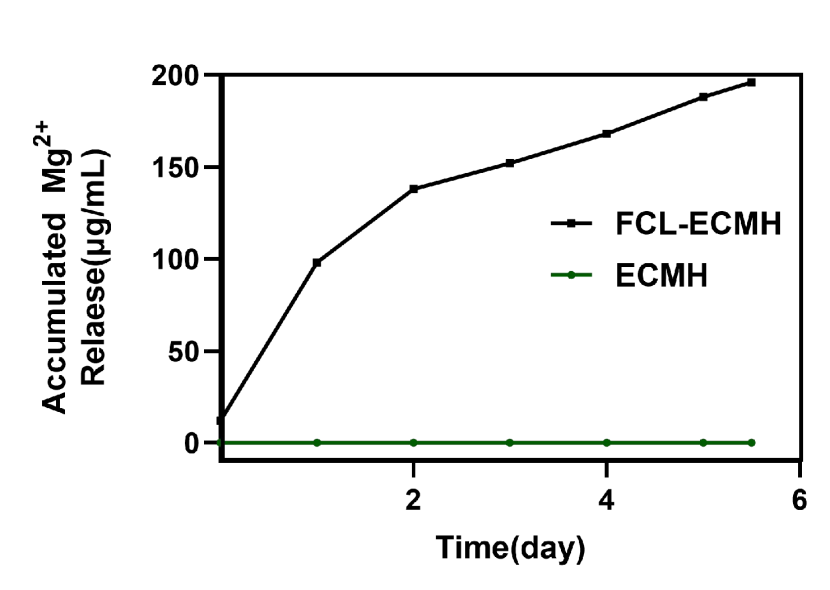


**Figure S7.** There is a moderate rate of simultaneous magnesium ions release from the FCL-ECMH (mean ± SD, n = 3)


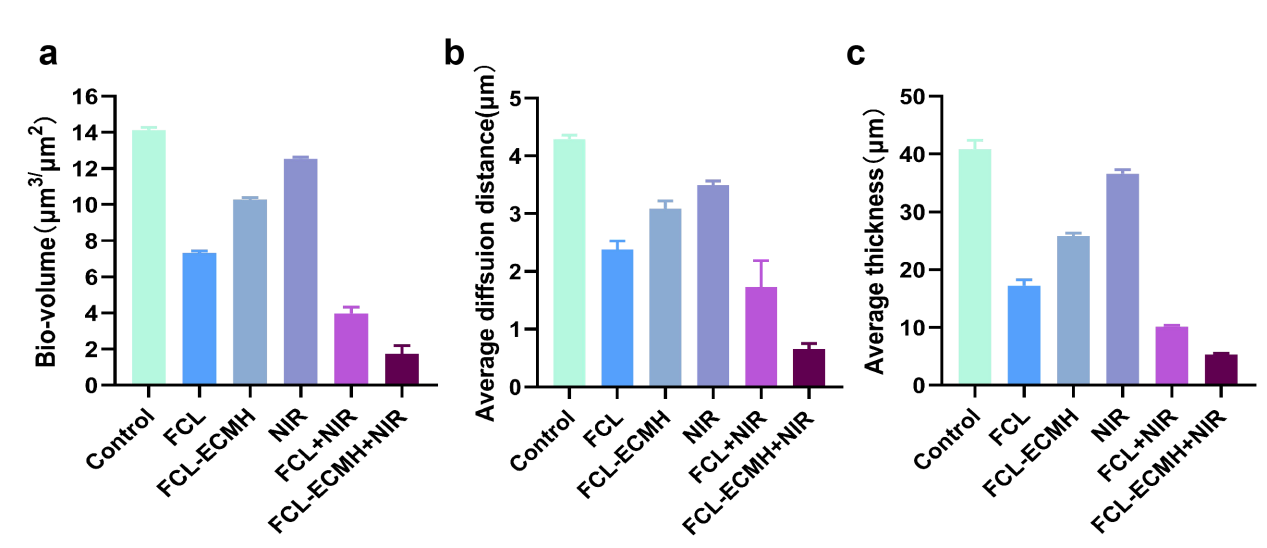


**Figure S8.** COMSTAT analysis of biofilms of different groups.


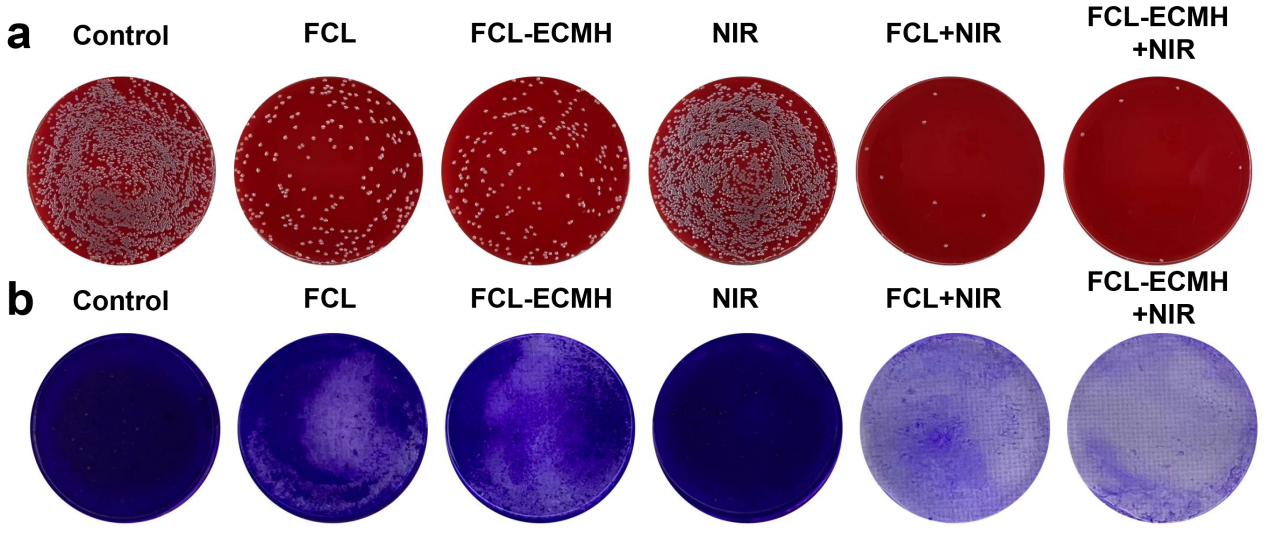


**Figure S9.** (a) Colony-forming units on an blood agar plates of *E. coli* after various treatment. (b) The photographs of *E. coli* biofilm processed with different treatments and subjected to crystal violet staining.


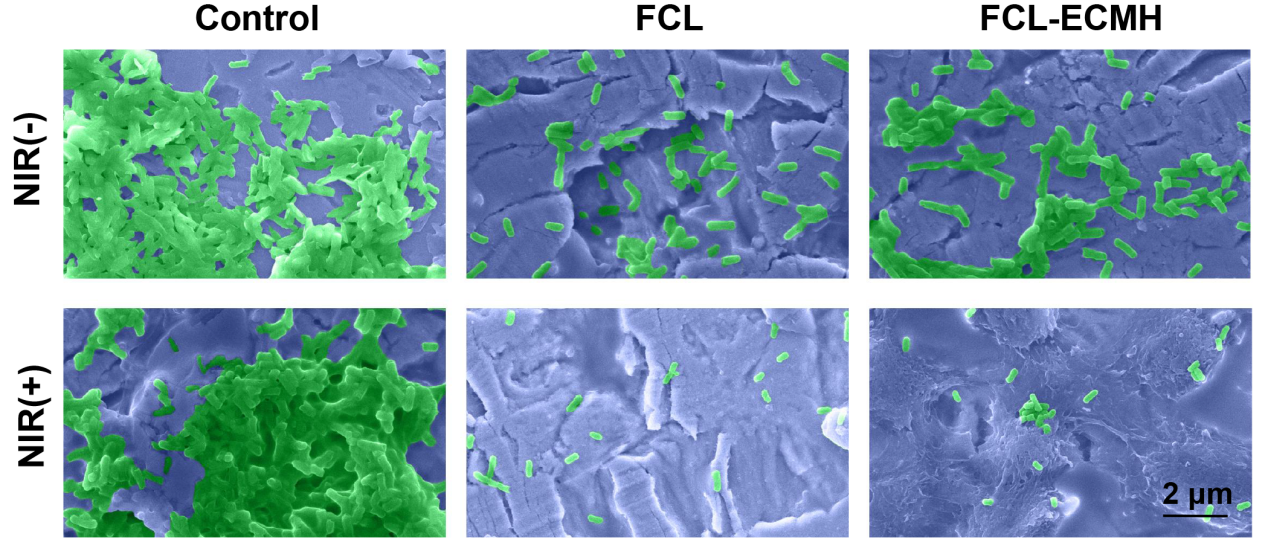


**Figure S10.** SEM images of *E. coli* biofilms. Scale bar, 2 μm.


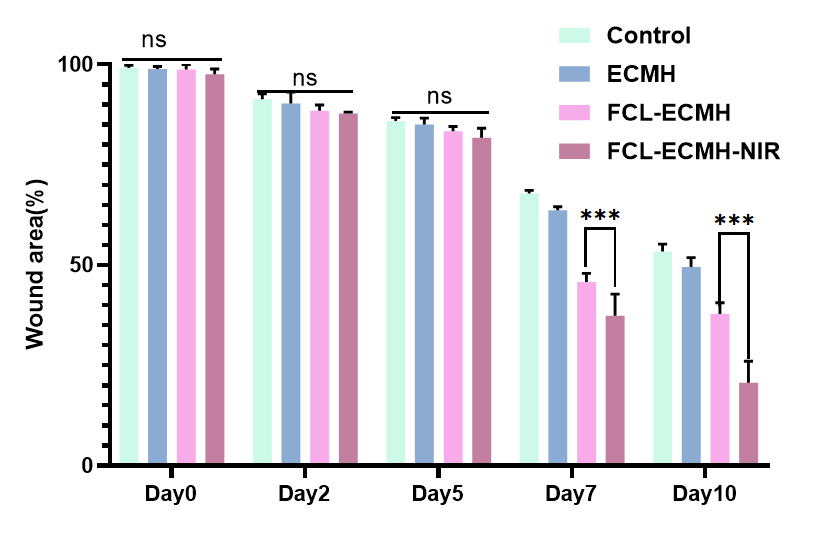


**Figure S11.** Statistics of wound closure rate in different groups (n =3). ***p < 0.001, and ns = not significant.

**Figure S12.** The quantitative analysis of CD31 based on immunohistochemical staining. ***p < 0.001


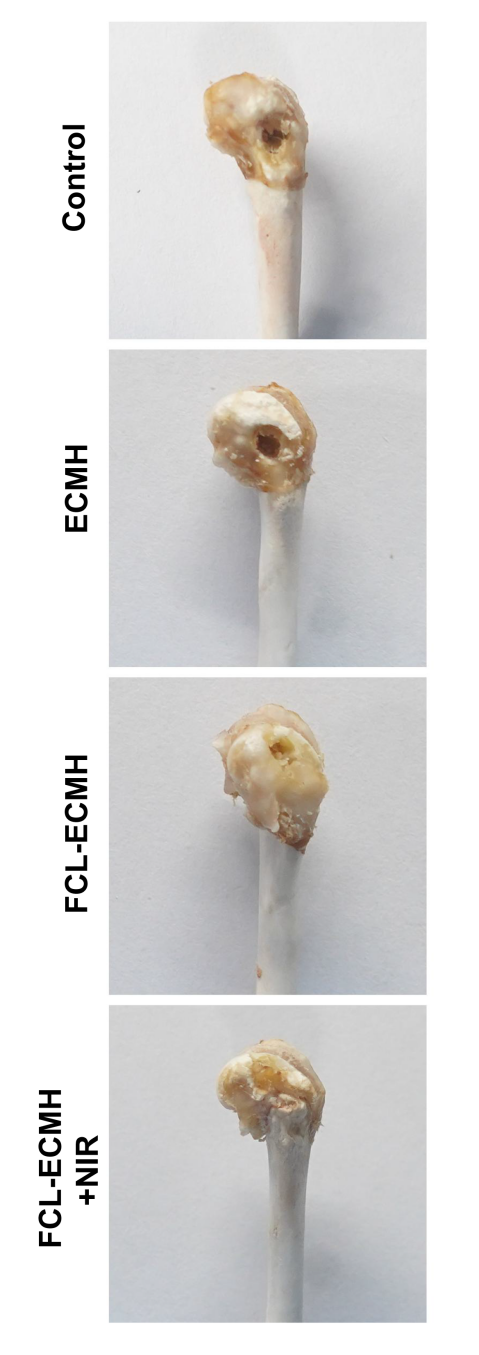


**Figure S13.** Following treatment, digital photos of femurs in different groups of rats.


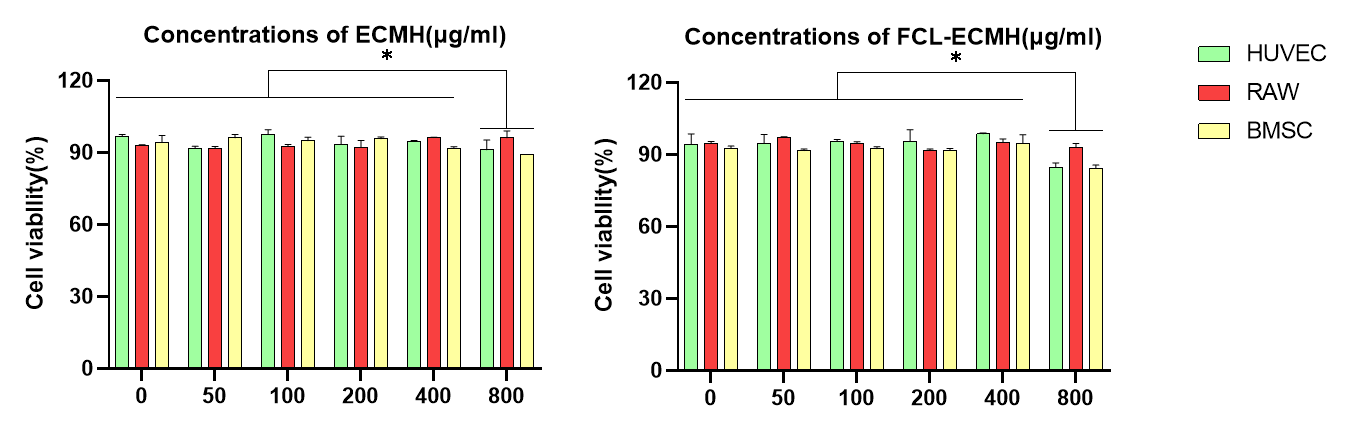


**Figure S14.** ECMH and FCL-ECMH cultures of HUVEC, RAW264.7, and BMSC cells assessed by CCK8 assay . *p < 0.1


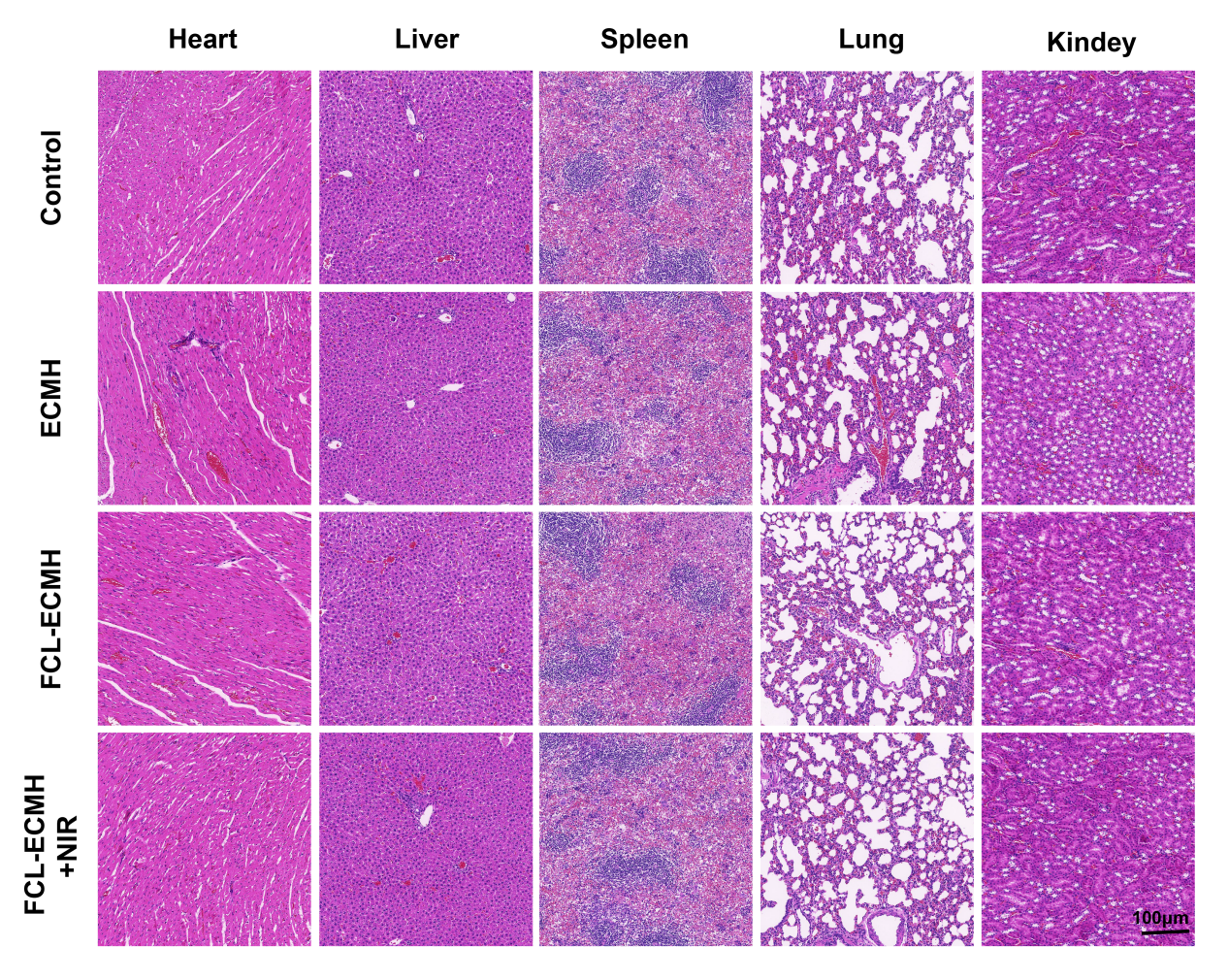


**Figure S15.** Heart, liver, spleen, lung, and kidney of rats stained with H&E. Scale bar, 100 μm .

**Supplementary References**

[1] a)Z. Zhang, B Jia, H Yang, Y Han, Q Wu, K Dai , Y Zheng, Biomaterials. 2021 Aug;275:120905; b)L. Zheng, D. Zhou, F. Ju, Z. Liu, C. Yan, Z. Dong, S. Chen, L. Deng, S. Chan, J. Deng, X. Zhang, Adv Sci (Weinh) 2023, 10, e2204592.
